# Malpositioned olecranon fracture tension-band wiring results in proximal radioulnar synostosis

**DOI:** 10.1186/s40001-015-0184-7

**Published:** 2015-10-29

**Authors:** Lukas Willinger, Martin Lucke, Moritz Crönlein, Gunther H. Sandmann, Peter Biberthaler, Sebastian Siebenlist

**Affiliations:** Department of Trauma Surgery, Klinikum rechts der Isar, Technical University Munich, Ismaningerstr. 22, 81675 Munich, Germany

**Keywords:** Olecranon fracture, Tension-band wiring, Radioulnar synostosis, Complication, Radiotherapy

## Abstract

**Background:**

Tension-band wiring (TBW) is a well-established fixation technique for two-part, transverse fracture types of the olecranon. However, complication rates up to 80 % are reported. By reporting on the enormous impact on the patient if failed the aim of the present report was to emphasize the importance of correct K wire positioning in TBW.

**Case presentation:**

We present the case of a 49-year-old woman who suffered from a radioulnar synostosis of the forearm due to malpositioned K wires after TBW treatment. The patient was treated by heterotopic bone resection supported by ossification prophylaxis (radiotherapy and Indomethacin). At follow-up of 12 months after revision surgery, elbow motion was unrestricted with a strength grade 5/5. The patient was free of pain and reported no restrictions in daily as well as sporting activities. Radiologic assessment showed no recurrence of heterotopic bone tissue.

**Conclusion:**

Intraoperative radiographic and clinical examination of the elbow is highly recommended to identify incorrect hardware positioning and, therefore, to avoid serious postoperative complications in TBW.

## Background

Isolated olecranon fractures are the most common fractures of the proximal ulna and account for 10 % of upper extremity fractures. The direct fall onto the elbow is the main injury pattern [1, 2]. Surgical treatment depends on the grade of fracture dislocation and the number of bony fragments [3]. Tension-band wiring (TBW) is a well-established fixation technique for two-part, transverse fracture types of the olecranon with sufficient bone quality [3, 4]. Even though TBW is often considered as an easy and convenient surgical procedure, several imperfections are described in the current literature and complication rates up to 80 % are reported [5–8]. Proximal migration of Kirschner (K) wires followed by the necessity of early hardware removal is the most frequent complication in case K wires are inserted into the medullary canal of the distal ulna fragment [5]. To avoid this complication, the modified technique involving the transcortical K wire placement through the anterior cortex of the ulna has been introduced [9, 10]. Nevertheless, extensive hardware protrusion through the anterior ulnar cortex may result in nerve and/or vascular injuries, impaired range of elbow motion and heterotopic ossification (HO) [11–15].

In the following case, we refer a proximal radioulnar synostosis (RUS) following TBW of a simple transverse olecranon fracture. To our research of the literature, there are just two articles on this topic, and only one of them reported about the therapeutic management of this severe complication [15, 16]. The purpose of the present article is, therefore, to emphasize the importance of correct K wire insertion during the TBW procedure by reporting on the enormous impact on the patient if failed. The surgical approach including pre- and postoperative measures is described in detail and discussed with reference to the current literature.

## Case presentation

**Fig. 1:**
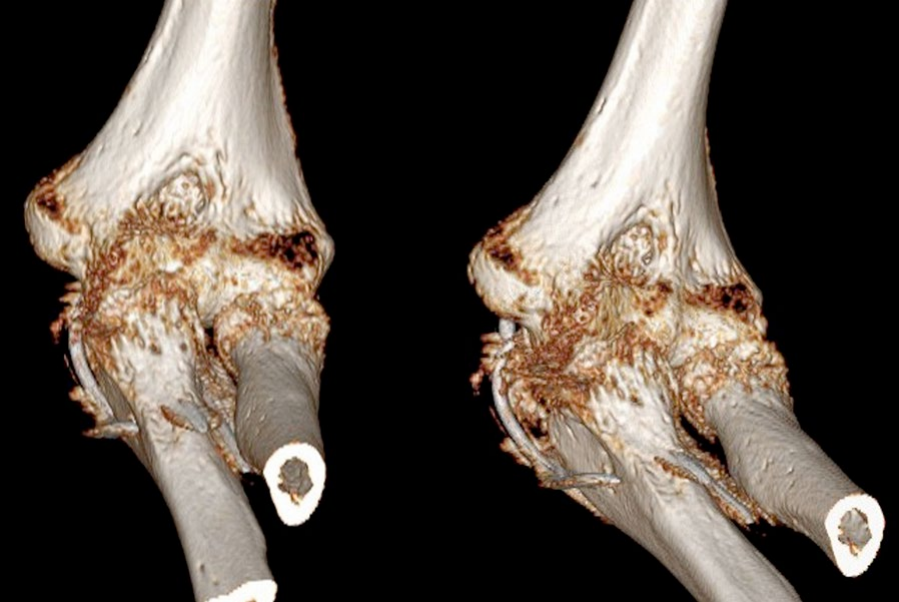
Malpositioned K wires after olecranon TBW. 3D computed tomography scans show the K wire placement within the radioulnar space at the radial tuberosity 6 weeks after initial surgery

A 49-year-old woman sustained a two-part olecranon fracture (AO 21-B1, Mayo type IIA [17]) in her left elbow. She was consequently treated by open reduction and internal fixation using the transcortical TBW technique. Immediately after surgery the patient complained about persistent pain in her elbow, wrist and shoulder as well as strong difficulties to rotate the forearm during physiotherapy. Despite the reported complaints the patient was advised to intensify physiotherapy. Follow-up radiographs 6 weeks postoperatively revealed not only union of the olecranon fracture, but also extensive protrusion of the K wires through the anterior ulnar cortex scratching the radial tuberosity (Fig. 1). Both K wires were consequently removed in a second operation, but the tension-band cerclage remained at its position. After K wires removal, the patient was encouraged again to enforce physiotherapy.

**Fig. 2:**
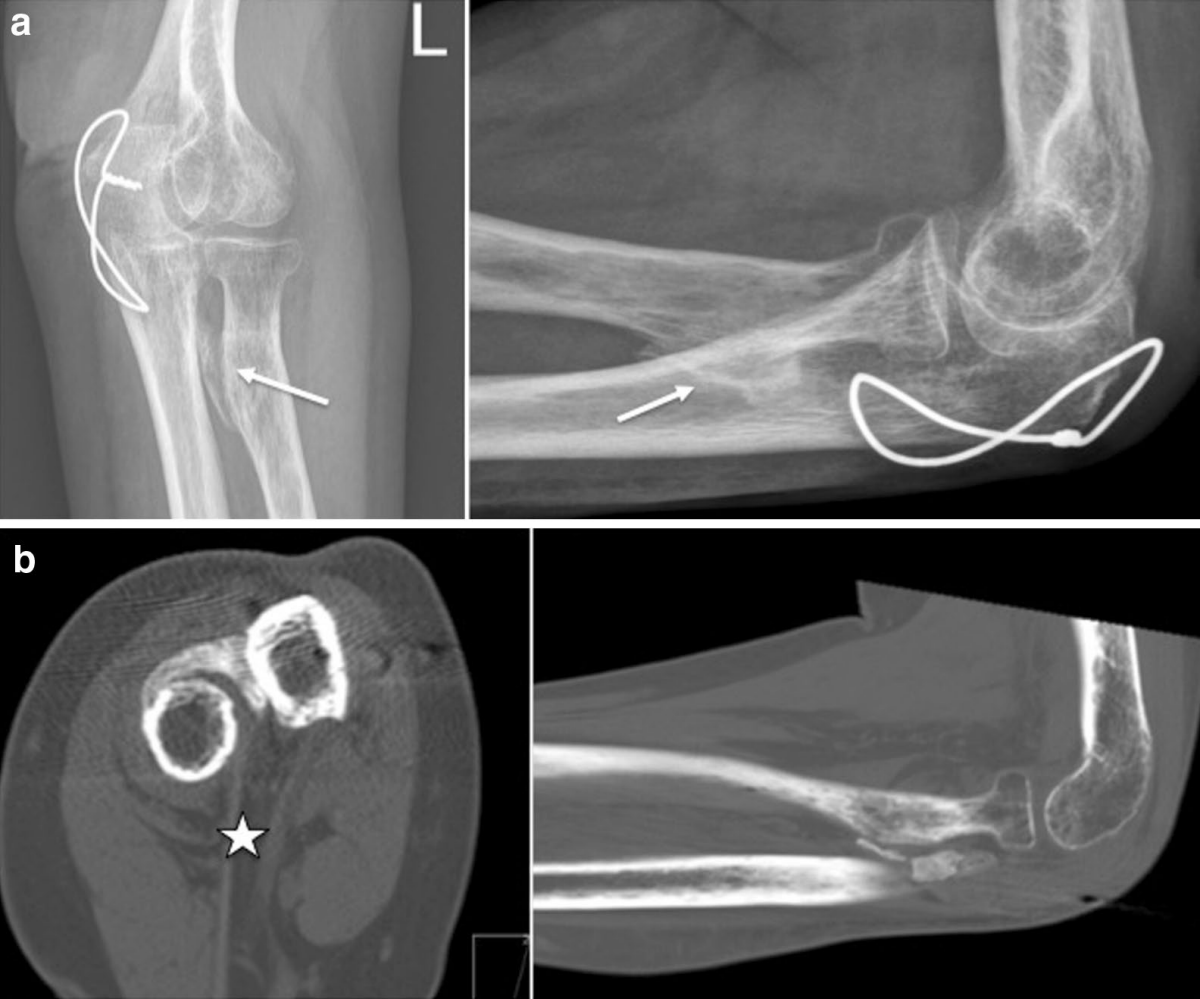
Proximal radioulnar synostosis. **a** Massive radioulnar synostosis (*white arrows*) at the level of the radial tuberosity 3 months after initial surgery. The olecranon fracture is healed while the cerclage wire remained in situ. **b** CT scans show the heterotopic ossification starting from the distal biceps tendon (*white star*) insertion to the ulnar cortex

Three months after trauma, the patient was presented to our outpatient clinic due to a total block of supination and pronation with the forearm fixed in neutral position. The extension–flexion arc of the elbow was 0°–5°–110°. Wrist and shoulder examinations showed a free range of motion (ROM); pain or sensomotoric restrictions did not exist. Radiographs showed a massive synostosis between the radial tuberosity and the proximal ulna where previously the K wires had penetrated the anterior radial cortex of the ulna (Fig. 2). Based on clinical and radiographic findings the resection of the synostotic bone was indicated to restore forearm motion.

**Fig. 3:**
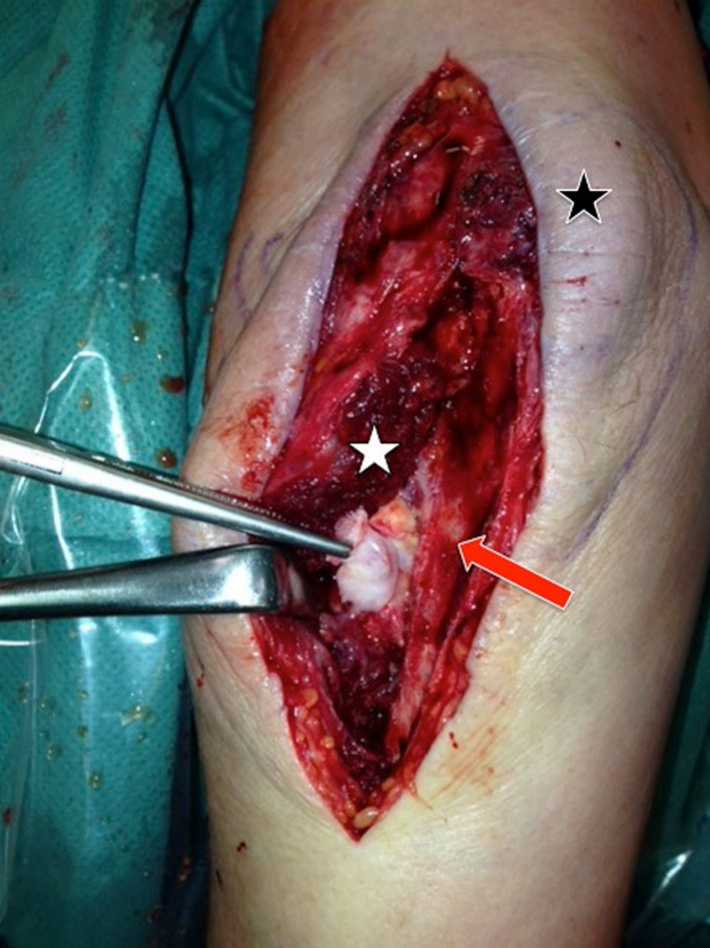
Dorsoradial approach for revision. Dorsoradial approach (*black star* olecranon tip) in prone position. After subperiosteal en bloc detachment of the supinator muscle (*white star*) from the proximal ulna (*red arrow*) the heterotopic bone formation (forceps) was exposed

Two hours before revision surgery, a prophylactic radiotherapy (with a dosage of 7 Gy) focused on the proximal aspect of the forearm was performed to minimize the risk of synostotic recurrence [18, 19]. The previously used dorsal approach was reopened and the remaining cerclage wire was removed a priori. Next, the approach was extended distally and the supinator muscle was subperiosteally detached en bloc from the proximal ulna to expose the heterotopic bone formation (Fig. 3). We decided for that approach to protect the posterior interosseous nerve (PIN) running through the supinator mass. The synostotic bone was first exposed from its proximal to its distal extent. Second, by subperiosteal preparation the heterotopic bone formation was dissected step-by-step to its radial extent at the radial tuberosity. This preparation was mainly performed by chiseling along well attached to the bone. Thereby, it was carefully observed not to damage the distal biceps tendon that was immured by heterotopic bone. The synostosis was finally removed all in one. Subsequent intraoperative examination revealed full pronation and supination movements before closure. For rehabilitation, active and passive elbow exercises including immediate free forearm rotation started under physiotherapist’s supervision the day after surgery. No splint or cast was applied. Indomethacin (50 mg 1–0–1 per day) was prescribed for 2 weeks for prophylaxis of ossification [20].

**Fig. 4:**
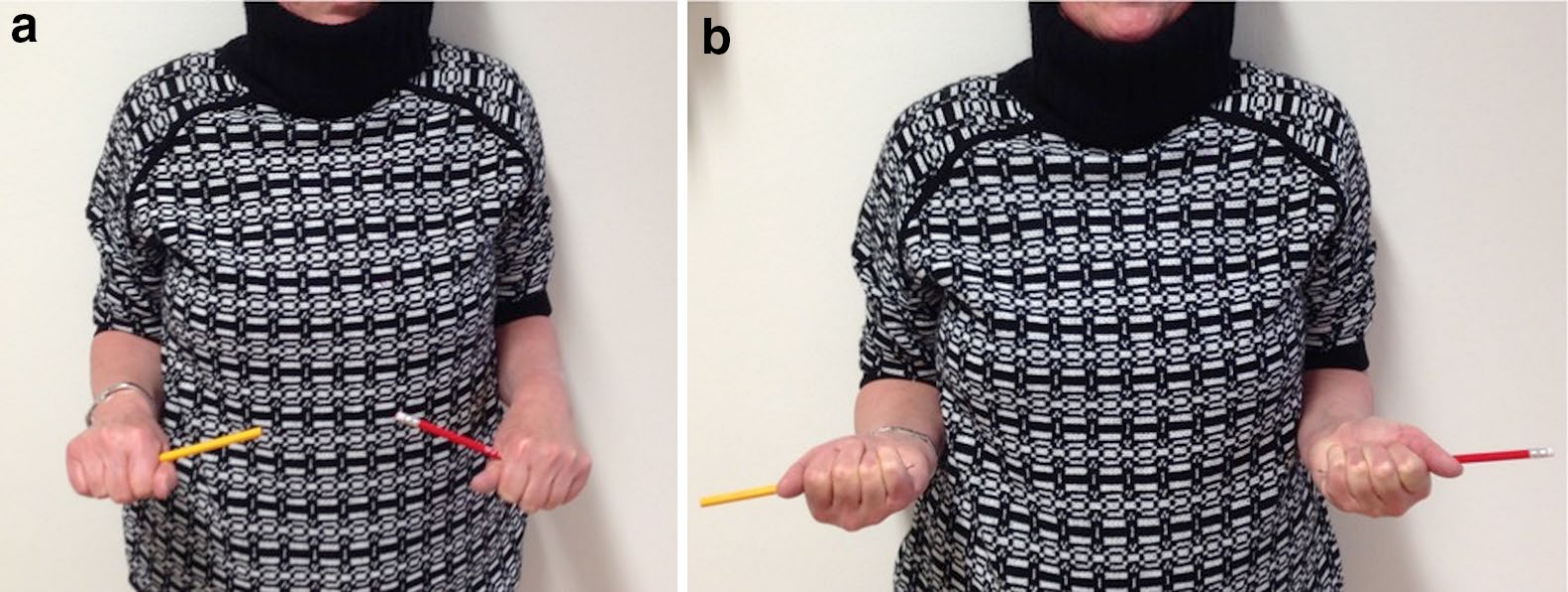
Unrestricted elbow function 12 months after revision surgery. **a** Unrestricted pronation and **b** supination in both arms (*left* elbow, *red stick*)

Ten weeks postoperatively the ROM of the elbow was 0°–10°–140° for extension–flexion arc and 75°–0°–90° for pronation–supination arc. At final follow-up 12 months after surgery, the ROM was unrestricted when compared to the uninjured arm side (Fig. 4). Supination strength was also unlimited presenting grade 5/5 on both arms. The patient was free of pain and reported no restrictions in daily as well as sporting activities (swimming three times per week). Radiologic assessment showed no recurrence of heterotopic bone tissue.

## Discussion and conclusion

TBW is often considered an easy-to-use operation technique to treat isolated simple olecranon fractures. Many authors showed good-to-excellent short- to long-term clinical outcomes [21–25], however, different complication rates were observed in the literature [1, 7, 11–16, 26, 27].

However, the development of an RUS due to hardware protrusion was—to the best of our knowledge—reported in only two articles so far [15, 16]. Velkes et al. [15] presented two cases of RUS following the transcortical technique within 3 months after surgery. The authors supposed as mechanism that the RUS was induced by soft tissue trauma and bleeding resulting from the K wire protrusion into the interosseous membrane and its surroundings. Consequently, secondary haematoma calcification was assumed for RUS development [28, 29]. Because both fractures showed bony healing within 6 weeks, a fracture-related mechanism for the RUS was not proposed by the authors. In contrast, De Carli et al. reported on a case of HO following transcortical malpositioned K wires abutting against the radial cortex [16].

In the present case, the synostotic bone formation also originated probably from the radius, particularly in that area where the K wires have been adjacent to the periosteum. Even though the K wires were removed 6 weeks after index surgery, signs of HOs at the proximal radioulnar joint were already visible (Fig. 2). When compared to other reported cases of RUS, the development of HO in the present case was very fast (probably induced from K wire irritation during forearm rotation within the first 6 weeks). We assume that the ongoing osseous erosion induced an inflammatory process with local release of growth factors (e.g., TGF-β) which contributes to the excessive bone proliferation process [30]. The reported persistent pain during physiotherapy might also be indicative for that suggestion. And additionally, soft tissue trauma during physiotherapy (e.g., of the supinator muscle) might have played a role in HO synthesis [31].

Regarding the clinical outcome, De Carli et al. [16] reported an excellent result after surgical removal of the synostosis and subsequent ossification prophylaxis with Indomethacin after 3 years of follow-up. Radiographs showed no signs of recurrence [16]. Similarly, with a 12 month follow-up the present patient showed excellent functional results and no radiographic signs of recurrent ossification as well. Velkes et al. [15] did not report further therapeutic measures because both patients declined revision.

The development of an RUS has been considered from soft tissue hematoma calcification by several authors [15, 28]. In the context of the present case we do not confirm this theory. The HO resection procedure would have also caused perioperative hematoma but no evidence of recurrent ossification was seen in the postoperative course. To avoid synostotic recurrence, different measures are frequently discussed in the current literature. In contrast to the case reports cited above, additional radiotherapy was performed in the present case before surgery. For lower extremities, it is evident that the combination of radiation and NSAID (non-steroidal anti-inflammatory drugs) should be used for prophylaxis of synostotic recurrence [20]. In upper extremity HOs likewise, a combined therapy regime has shown to successfully prevent synostotic recurrence [18, 32]. HOs around the elbow is not suspected to appear later than 3 months after the initiating trigger (trauma or surgery) [18, 33]. De Carli et al. [16] applied Indomethacin over the period of 6 weeks postoperatively in their case. Their decision was based on the concomitant nonunion of the olecranon fracture and the higher risk of non-healing subsequent to radiotherapy [16, 34]. After intramedullary repositioning of the K wires and excision of HO with osteoperiosteal decortication, fracture union was achieved [16]. The authors supposed that the malpositioned K wires and subsequent RUS might play a role during development of the nonunion by allowing rotational movement to the fracture site [16]. The present olecranon fracture showed bony healing within 6 weeks despite the malpositioned K wires and an impingement at the proximal radius, respectively. Therefore, one might speculate that the synostotic bone development in the present case was not induced due to a mechanical impingement phenomenon resulting in loosened K wires and fracture nonunion, but due to ongoing soft tissue irritation and periosteal microtrauma at the proximal radius. De Carli et al. [16], in their case report, also assumed that the HO developed as a result of periosteal stimulation following K wire impingement during pronation and supination.

**Fig. 5:**
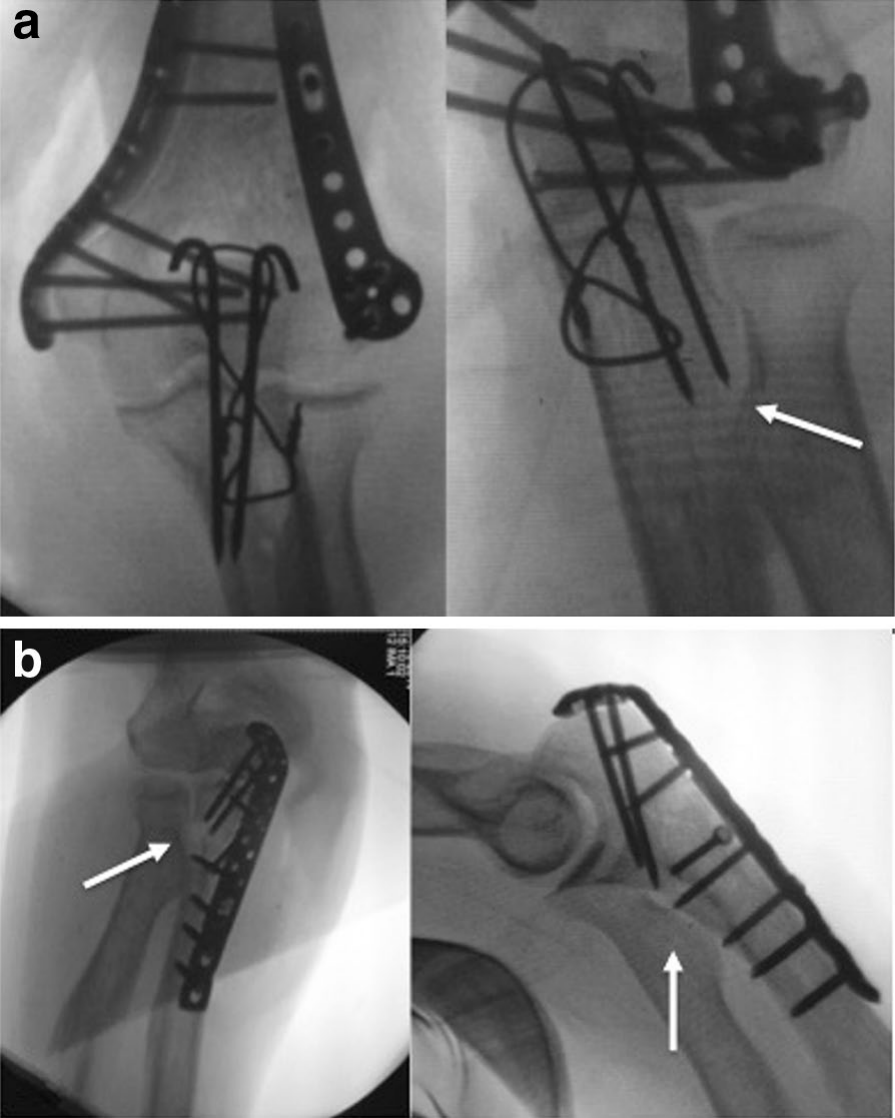
Intraoperative fluoroscopic examination. **a** TBW after olecranon osteotomy for osteosynthesis of distal humeral fracture (*left* K wires are directed ulnarly, *right* the slight oblique a.p. view verifies the free proximal radioulnar space (*white arrow*); **b** both views (*white arrows*) demonstrate the free proximal radioulnar space after plating of the proximal ulna

To prevent such intraoperative complications and not to harm proximate structures, the explicit knowledge of the proximal forearm anatomy is required. For TBW, current studies recommend to insert the K wires from a more lateral entry point into the olecranon tip and aiming towards the ulnar midshaft to avoid interference with the proximal radial shaft and the proximal radioulnar articulation, respectively [11, 27]. Surgeons have to be aware of the normal varus angulation of the proximal ulna not to place the pins in direction of the radius and to avoid impingement between hardware and radial neck [35, 36]. While K wires are inserted, the forearm should be positioned in supination [11]. Intraoperative fluoroscopic examination is essential to recognize implant interfering with the proximal radius. For TBW and for plating of olecranon fractures as well, a clear proximal radioulnar space (PRUJ, respectively) has to be outlined using a slightly oblique a.p. view during operative procedure (Fig. 5). In addition, an accurate intraoperative assessment of forearm rotation should be performed to ensure free ROM and the absence of noticeable crepitation.

 Impaired rotational movements during rehabilitation have led one to think about interfering pins. In case of inexplicable discomfort and ongoing pain, further radiological assessment (favored CT scans) is indicated because a malpositioning of K wires is often not visible in standard X-rays (a.p. and lateral view). To sum up, accurate operative approach is necessary to avoid such perooperative adverse events in olecranon TBW as described above.

In the present case clinical outcome was excellent after HO resection with supportive radiotherapy and Indomethacin administration. No HO recurrence was detected throughout which might confirm the theory that the RUS was induced due to periostal K wire scratching during forearm rotation resulting in local inflammatory process but not from postoperative hematoma calcification.

## Consent

Written informed consent was obtained from the patient for publication of this case report and any accompanying images. A copy of the written consent is available for review by the Editor-in-Chief of this journal.

## Abbreviations

HO: heterotopic ossification
K: Kirschner
PIN: posterior interosseous nerve
ROM: range of motion
RUS: radioulnar synostosis
TBW: tension-band wiring
